# Identification of the fatty acid synthase interaction network via iTRAQ-based proteomics indicates the potential molecular mechanisms of liver cancer metastasis

**DOI:** 10.1186/s12935-020-01409-2

**Published:** 2020-07-21

**Authors:** Juan Huang, Yao Tang, Xiaoqin Zou, Yi Lu, Sha She, Wenyue Zhang, Hong Ren, Yixuan Yang, Huaidong Hu

**Affiliations:** 1 Institute for Viral Hepatitis, Key Laboratory of Molecular Biology for Infectious Diseases (Ministry of Education), Department of Infectious Diseases, The Second Affiliated Hospital, Chongqing Medical University, Chongqing, 400016 People’s Republic of China; 2 The Second Affiliated Hospital of Chongqing Medical University, 74 Linjiang Road, Chongqing, 400010 China

**Keywords:** Fatty acid synthase, Liver cancer, Metastasis, Protein–protein interaction, Isobaric tags for relative and absolutely quantitation-based proteomics

## Abstract

**Background:**

Fatty acid synthase (FASN) is highly expressed in various types of cancer and has an important role in carcinogenesis and metastasis. To clarify the mechanisms of FASN in liver cancer invasion and metastasis, the FASN protein interaction network in liver cancer was identified by targeted proteomic analysis.

**Methods:**

Wound healing and Transwell assays was performed to observe the effect of FASN during migration and invasion in liver cancer. Isobaric tags for relative and absolute quantitation (iTRAQ)-based mass spectrometry were used to identify proteins interacting with FASN in HepG2 cells. Differential expressed proteins were validated by co-immunoprecipitation, western blot analyses and confocal microscopy. Western blot and reverse transcription-quantitative polymerase chain reaction (RT-qPCR) were performed to demonstrate the mechanism of FASN regulating metastasis.

**Results:**

FASN knockdown inhibited migration and invasion of HepG2 and SMMC7721 cells. A total of, 79 proteins interacting with FASN were identified. Additionally, gene ontology term enrichment analysis indicated that the majority of biological regulation and cellular processes that the FASN-interacting proteins were associated with. Co-precipitation and co-localization of FASN with fascin actin-bundling protein 1 (FSCN1), signal-induced proliferation-associated 1 (SIPA1), spectrin β, non-erythrocytic 1 (SPTBN1) and CD59 were evaluated. Knockdown of FASN in liver cancer reduced the expression of FSCN1, SIPA1, SPTBN1 and CD59. Furthermore, inhibition of FASN, FSCN1 or SPTBN1 expression in liver cancer resulted in alterations of epithelial–mesenchymal transition (EMT)-associated markers E-cadherin, N-cadherin, vimentin and transcription factors, Snail and Twist, at the mRNA level, and changes in matrix metallopeptidase (MMP)-2 and MMP-9 protein expression.

**Conclusion:**

The results suggested that the FASN-interacting protein network produced by iTRAQ-based proteomic analyses may be involved in regulating invasion and metastasis in liver cancer by influencing EMT and the function of MMPs.

## Background

Cancer is a leading cause of mortality in economically developed countries and developing countries. Worldwide, the incidence of liver cancer is increasing and the increase is likely to continue for a number of decades [1]. Liver cancer remains the fifth most common malignant tumor in men and the seventh most common among women, worldwide, and is the third leading cause of cancer-associated mortality, exceeded only by stomach and lung cancer [2, 3]. The most common risk factors for liver cancer are chronic hepatitis B and C infection, long-term alcohol abuse, dietary exposure to aflatoxin B1, smoking and several metabolic diseases [4]. Liver cancer has an extremely high recurrence rate with poor prognoses, mainly due to active angiogenesis, a high propensity to metastasize and the rapid proliferation of tumor cells [5]. Metastasis is considered a sign of deterioration and the major cause of morality for patients with liver cancer [6]. During the metastatic process, cancer cells undergo detachment, migration, invasion and adhesion. Tumor metastasis involves a series of sequential and interconnected steps, commonly referred to as the ‘invasion-metastasis cascade’. Effective treatment of metastatic liver cancer is limited, due to a lack of understanding of the mechanisms underlying the metastatic process [7, 8]. In order to develop effective therapeutic strategies, there is an urgent need to investigate the molecular basis of liver cancer metastasis.

Due to the confirmed value of quantitative proteomics, efforts have been made to develop and improve quantitative methods. In recent years, isobaric tags for relative and absolute quantification (iTRAQ)-based mass spectrometry (MS) quantification methods have become powerful tools to quantify differentially expressed proteins (DEPs) and identify protein-interaction networks [9]. As opposed to the classic proteomic quantification methods, using dyes, fluorophores or radioactivity, the iTRAQ-based MS techniques can be used for high-throughput analyses, and have a wide range of separation, high accuracy and repeatability [9]. Additionally, the technique can facilitate the simultaneous analysis of up to eight samples in one experiment, precisely identifying and quantifying thousands of proteins from complex samples. These benefits have led to iTRAQ proteomic methods increasing in popularity over the past 5 years [10].

Fatty acid synthase (FASN), a key enzyme required for the synthesis of fatty acids and precursors of certain biologically important lipids, is the most well-investigated lipogenic protein in cancer research [11]. FASN is highly expressed in various types of cancer, and is closely associated with tumor stage and prognosis in breast, prostate and gastric cancer [12–14]. Several studies have demonstrated that FASN is associated with the activation of various oncogenic signaling pathways, including the phosphoinositide 3-kinase (PI3K)/Akt serine/threonine kinase, Wnt/β-catenin signaling and transforming growth factor-β (TGF-β)-induced epithelial–mesenchymal transition (EMT) pathways [15–17]. Therefore, FASN is a potential molecular target for cancer treatment. Our previous study demonstrated that FASN was overexpressed in liver cancer tissues and cells, and the data indicated that FASN may be closely associated with liver cancer metastasis [18]; however, the underlying molecular mechanism of FASN in liver cancer metastasis has not yet been identified.

With the advances in proteomics research, an increasing number of studies have demonstrated that protein–protein interactions have a key role in the pathogenesis of malignant tumors by regulating numerous biological processes in cells [19]. The interacting proteins are rapidly and specifically identified by the coupling of MS technologies with co-immunoprecipitation (co-IP), providing a rapid, sensitive and reliable approach for discovering and identifying protein interactors [20]. To clarify the molecular mechanism of FASN in liver cancer metastasis, FASN-interacting proteins were investigated using a targeted proteomics approach (co-IP coupled with iTRAQ-based MS), and the molecular functions and biological processes of proteins interacting with FASN were analyzed using bioinformatics methods. The identification of the protein complexes will provide an increased understanding of the FASN interactome, and has the potential to elucidate the molecular mechanisms involved in liver cancer invasion and metastasis.

## Methods

### Reagents

iTRAQ eight-plex kits were purchased from Applied Biosystems (Thermo Fisher Scientific, Inc., Waltham, MA, USA). All electrophoresis reagents were acquired from Bio-Rad Laboratories, Inc. (Hercules, CA, USA). Horseradish peroxidase (HRP)-conjugated immunoglobulin G (IgG) antibodies (goat anti-mouse and goat anti-rabbit), and polyvinylidene fluoride (PVDF) membranes were obtained from GE Healthcare (Chicago, IL, USA). Monoclonal or polyclonal antibodies against FASN, fascin actin-bundling protein 1 (FSCN1), signal-induced proliferation-associated 1 (SIPA1), spectrin β, non-erythrocytic 1 (SPTBN1), CD59, matrix metallopeptidase (MMP)-2 and MMP-9 were acquired from Abcam (Cambridge, MA, USA). β-actin was acquired from Santa Cruz Biotechnology, Inc. (Dallas, TX, USA). Small interfering RNA (siRNA) against FASN (ID nos. HSS103565 and HSS176712), FSCN1 (ID no. HSS110044) and SPTBN1 (ID no. HSS110164), a negative control (ID no. 12935-400) and Lipofectamine^®^ 2000 were purchased from Invitrogen (Thermo Fisher Scientific, Inc.). Protein A/G Beads were acquired from GE Healthcare. IP lysate buffer was purchased from Beyotime Institute of Biotechnology (Haimen, China). Opti-MEM and fetal bovine serum (FBS) were purchased from Gibco (Thermo Fisher Scientific, Inc.).

### Cell culture and FASN, FSCN1 or SPTBN1 siRNA transfection

Human liver cancer cell lines HepG2 and SMMC7721 were obtained from the Chinese Academy of Sciences (Shanghai, China), and these cells were periodically subjected to model certification. The cell lines were cultured in high-glucose Dulbecco’s modified Eagle’s medium (DMEM; supplemented with 2 mM glutamine, 0.1% nonessential amino acids, 1.0 mM sodium pyruvate, 100 IU/ml penicillin and 100 μg/ml streptomycin) supplemented with 10% FBS at 37 °C and 5% CO_2_.

HepG2 and SMMC7721 cells were transfected with FASN-specific siRNA, FSCN1-specific siRNA, SPTBN1-specific siRNA, blank control or a negative control siRNA using Lipofectamine^®^ 2000 and OPTI-MEM (Gibco; Thermo Fisher Scientific, Inc.). Following transfection, cells were cultured in high-glucose DMEM without serum for 4 h, and the media was subsequently replaced with DMEM supplemented with 10% FBS for continued cultivation.

### Wound healing and Transwell assays

HepG2 and SMCC7721 cells transfected with FASN-specific siRNA or control siRNA in Wound healing. These two cells lines were transfected with FASN-specific siRNA, blank control or control siRNA in Transwell assays. These cells were performed 2 days after transfection. When the cells were adherent and ~ 100% confluent in 6-well plates, a wound was created in the cell monolayer using a sterile P200 pipette tip, followed by three gentle washes with PBS to remove cellular debris. Cell migration was determined by the closure of the wounds, which were imaged at 0 and 24 h under a microscope. The Transwell invasion assay was performed using a 24-well Cell Invasion Assay kit (Cell Biolabs, Inc., Beijing, China). Viable cells (~ 1 × 10^5^) were loaded into the upper chambers, separated from the lower chambers by an 8-µm pore size membrane pre-coated with Matrigel (BD Biosciences, San Jose, CA, USA). Cells were cultured for 24 h and the invading cells attached underneath the chamber membrane were stained using cyQuant GR fluorescent dye and quantified at 560 nm. The knockdown of FASN was determined by western blot analysis. Each experiment was performed in triplicate.

### Protein sample preparation, co-IP and iTRAQ labeling

Cells transfected with FASN-specific siRNA or control siRNA were washed twice with PBS when ~ 80% confluent. Whole cell lysates were collected from HepG2 cells and SMMC7721 cells. To remove cellular debris, the cell lysate was centrifuged at 13,000×*g* for 20 min at 4 °C. A 2D Quantification kit (GE Healthcare) was used to detect protein concentration in the lysates. For co-IP, 1 mg extracted protein was incubated with 2 µg FASN antibody overnight at 4 °C with gentle agitation, followed by 2 h incubation with 20 µl Protein A/G agarose beads at 4 °C with gentle agitation. Prior to incubation, the beads were resuspended and washed three times with IP lysis buffer. The bead-antibody-antigen complex was then centrifuged at 4000×*g* for 5 min at 4 °C and the bead complex was washed three times with IP lysis buffer (the supernatant of the last collection as the input group samples). Bound proteins were eluted by heating the collected beads in SDS-PAGE loading buffer containing 10% β-mercaptoethanol for 5 min at 95 °C. The supernatant was used for western blot analysis. Control samples were obtained through the IP procedure with the elimination of primary antibody (bead group) or substituting IgG antibody for the primary antibody (IgG group). The eluted proteins were acetone-precipitated overnight at − 20 °C and re-dissolved in lysis buffer, and denatured and cysteine-blocked according to the iTRAQ manufacturer’s protocol. Following trypsin (Promega Corporation, Madison, WI, USA) digestion, the protein samples were labeled as follows: HepG2 cells without FASN knockdown, 114 and 117 tags; and HepG2 cells with FASN knockdown, 118 and 121 tags. For subsequent analysis, the iTRAQ-labeled samples were pooled.

### Fractionation of peptides

The pooled, labeled samples were solubilized in a Pharmalyte (GE Healthcare Life Sciences, Little Chalfont, UK) and urea solution, applied onto pre-hydrated immobilized pH gradient (IPG) strips (pH 3–10) and then focused successively at 68 kV/h on an IPGphor system (GE Healthcare Life Sciences). The peptides were subsequently extracted from the gels using a solution of formic acid and acetonitrile. Fractions were lyophilized in a vacuum concentrator and purified on SPE C18 columns (Supelco; Sigma-Aldrich, Darmstadt Germany). The purified fractions were re-lyophilized, and stored at − 20 °C prior to MS analysis.

### MS

The purified peptide fractions were resuspended in Buffer A (2% acetonitrile and 0.1% formic acid) and injected into a Nano LC ESI MS/MS system (SCIEX, Framingham, MA, USA). The peptides were separated on a C-18 PepMap column (75 μm × 15 cm; Dionex; Thermo Fisher Scientific, Inc.) at a flow rate of 0.3 μl/min using a solvent gradient of 2–100% Buffer B (98% acetonitrile and 0.1% formic acid). The peptides were ionized at an ion spray voltage of 2300 eV using a nanoelectrospray ionization source and analyzed by a Nano LC ESI MS/MS system. For data acquisition, the MS was set in positive ion mode and the mass range of 300–1800 m/z was used. The two most abundantly charged peptides > 20 counts were selected for MS/MS at a dynamic exclusion of 30 s ± 50 mDa.

Data was processed using ProteinPilot™ software (v2.0; SCIEX) and compared to the International Protein Index Human database (v3.77). Cysteine modified by methyl methanethiosulfate was designated as a fixed modification. For protein identification and quantitation, a strict set of criteria was formulated. Briefly, a selection threshold of protein score > 1.3, and at least two unique peptides with 95% confidence at a 5% false discovery rate were counted as significant [21–23].

### Bioinformatics analysis

The proteins and genes differentially expressed between the control siRNA group and FASN siRNA group were identified using ProteinPilot™. The gene ontology (GO) term enrichment analysis and generation of the hierarchical clustering heat map for the identified DEPs and DEGs were performed using PANTHER (www.pantherdb.org/tools/) and WebGestalt (www.webgestalt.org/) toolkits.

### Western blot analysis

IP samples were subjected to SDS-PAGE using 10% gels and subsequently transferred to PVDF membranes. The membranes were blocked with 5% non-fat dry milk in Tris-buffered saline solution with 0.1% Tween-20 (TBS-T) for 2 h at room temperature, and subsequently incubated with the primary antibodies (1:500–1:1000 dilution) in TBS-T buffer overnight at 4 °C. After washing with TBS-T buffer three times for 10 min, membranes were incubated with HRP-conjugated secondary antibodies (1:10,000 dilution) for 2 h at room temperature. The membranes were washed again with TBS-T following incubation and visualized using the ECL detection system (Bio-Rad Laboratories, Inc., Hercules, CA, USA). All the western blot analyses were performed at least three times.

### Confocal microscopy

HepG2 and SMMC7721 cells were plated in 35 mm confocal culture dishes for 48 h. The cells were rinsed with PBS three times and fixed with 4% paraformaldehyde for 30 min at room temperature. Following fixation, the cells were washed again with PBS and permeabilized with 0.2% Triton X-100 solution for 15 min at room temperature. The cells were blocked in 5% bovine serum albumin for 1 h at 37 °C, followed by incubation with primary antibody (1:50–1:100 dilution) for 18 h at 37 °C. On the following day, the cells were washed in PBS with 0.1% Tween-20 three times, then incubated with fluorescent-labeled secondary antibodies (Invitrogen; Thermo Fisher Scientific, Inc.) at 1:200 dilution in the dark for 3 h at 37 °C. DAPI (5%) diluted in methanol was used to counterstain nuclei for 15 min at room temperature in the dark. Finally, the cells were visualized using a laser scanning confocal microscopy (Nikon Corporation, Tokyo, Japan). Each experiment was performed in triplicate.

### Reverse transcription-quantitative polymerase chain reaction (RT-qPCR)

Total RNA was isolated using TRIzol reagent (Invitrogen; Thermo Fisher Scientific, Inc.) following the manufacturer’s protocol. First-strand cDNA was produced using a Reverse Transcription kit (Thermo Fisher Scientific, Inc.). A Fast PCR kit (KAPA SYBR; Kapa Biosystems; Roche Diagnostics, Basel, Switzerland) was used for qPCR with gene-specific primers to amplify E-cadherin (ID no. Hs00345541_CE), N-cadherin (ID no. Hs00258119_CE), vimentin (ID no. Hs00580303_CE), Snail (ID no. Hs00450570_CE), Twist (ID no. Hs00284538_CE) and GAPDH (ID no. Hs00115502_CE). Expression data were analyzed using the 2^−ΔΔCq^ method [24]. RT-qPCR analyses were repeated at least three times.

### Statistical analysis

Statistical analysis was performed by using SPSS software (v13.0; SPSS, Inc., Chicago, IL, USA). Continuous variables are presented as the mean ± standard deviation. Differences between groups were analyzed by the Student’s t-test or a Mann–Whitney U test. Qualitative variables are presented as counts and percentage, and were analyzed using the χ^2^ test. All statistical tests were bilateral and P < 0.05 was considered to indicate a statistically significant difference.

## Results

### Effect of FASN on cell migration and invasion in liver cancer

**Fig. 1:**
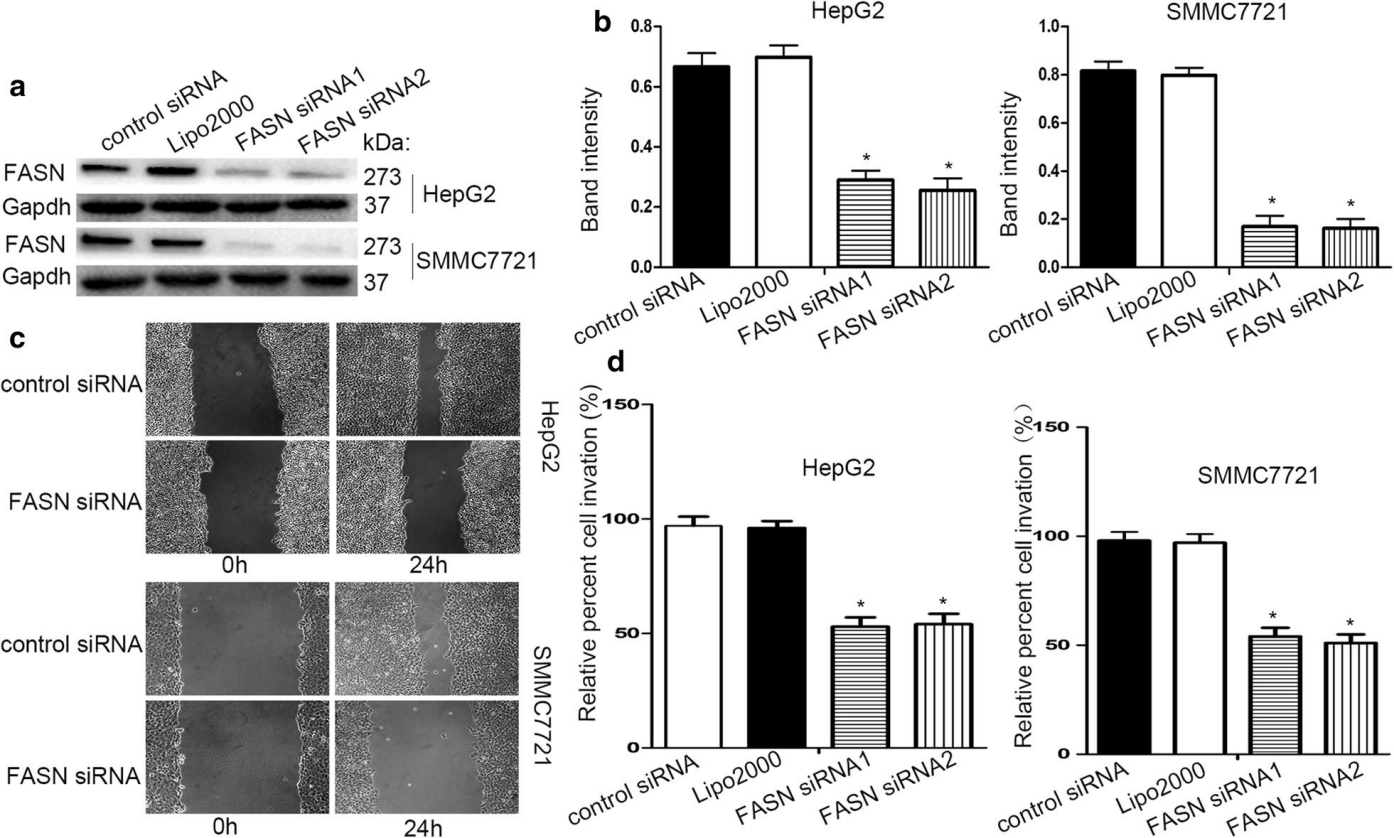
FASN has an important effect on the migration and invasion of liver cancer cells. **a**, **b** Western blot analysis demonstrated that knockdown of FASN significantly reduced FASN protein levels. **c** The migration ability of cells in the wound healing assays following FASN silencing (10× magnification). **d** The invasion ability of cells in the Transwell assays following FASN silencing. The band intensity analysis of protein levels was performed using GAPDH as reference. Each experiment was performed in triplicate. *P < 0.05. Values are presented as the mean ± standard deviation. *FASN* fatty acid synthase

To identify the effect of FASN during migration and invasion in liver cancer, a FASN-targeting siRNA was transfected into HepG2 and SMCC7721 cells. The FASN-specific siRNA effectively silenced FASN expression (Fig. 1a, b). Based on the wound healing assay, FASN knockdown significantly inhibited the migration ability of HepG2 and SMCC7721 cells (Fig. 1c). Additionally, in the Transwell assay, the invasion capability of HepG2 and SMCC7721 cells was significantly decreased by silencing of FASN (Fig. 1d).

### iTRAQ quantification of the FASN interactome

**Fig. 2:**
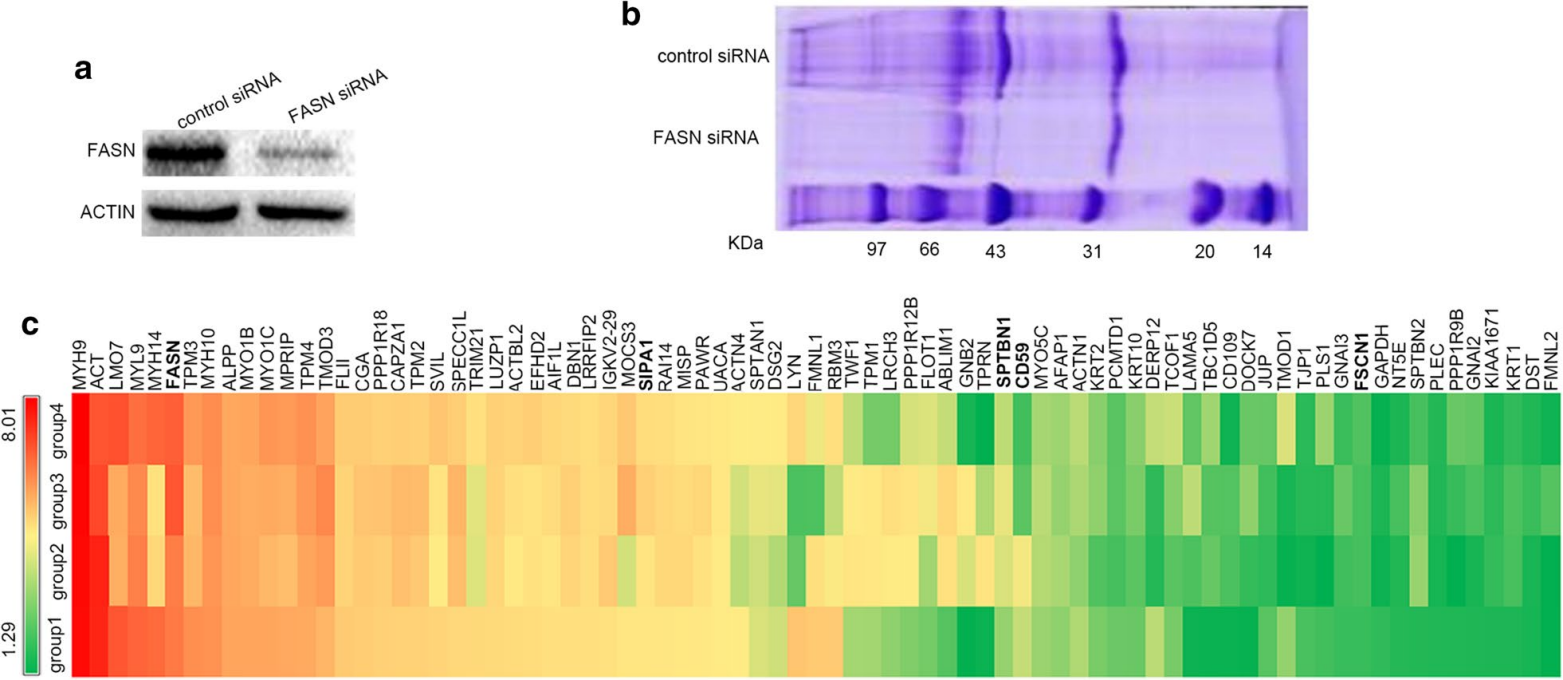
Silencing efficiency of FASN siRNA and heat map of proteins interacting with FASN. **a** HepG2 cells were transfected with FASN siRNA. Western blot analysis was used to determine the inhibition efficiency of FASN siRNA. **b** The differential strip of the control and FASN siRNA group evaluated by mass spectrometry. **c** The heat map of FASN and proteins interacting with FASN. The color scale indicates the fold change in protein expression in each group. 114:118 (group 1 and group 2) and 117:121 (group 3 and group 4) refer to the expression of FASN and proteins interacting with FASN after transfection with control siRNA and FASN siRNA in HepG2 cells. *FASN* fatty acid synthase, *siRNA* small interfering RNA

Co-IP and iTRAQ-based MS were coupled to identify proteins that interact with FASN. A flow chart of the iTRAQ method used is presented in Additional file 1: Fig. S1. HepG2 cells transfected with FASN-siRNA exhibited significantly downregulated of FASN expression (Fig. 2a). The proteins that were differentially expressed when comparing the control group and the siFASN-group are presented in Fig. 2b. A total of 79 unique DEPs were identified when comparing the control siRNA group and the FASN siRNA group (Table 1). The hierarchical clustering heat map of the differentially expressed proteins is presented in Fig. 2c.

**Table 1:** The proteins observed to be differentially expressed by iTRAQ analysis in HepG2 cells: control vs FASN knockdown

N	Accession	Gene symbol	Name	Peptides (95%)	114:118	PVal 114:118	117:121	PVal 117:121
1	sp|P35579|MYH9_HUMAN	MYH9	Myosin-9	1552	7.733154	1.60E−43	7.994438	0
2	sp|P35580|MYH10_HUMAN	MYH10	Myosin-10	467	4.294018	5.51E−30	4.44725	2.10E−29
3	sp|P49327|FAS_HUMAN	FASN	Fatty acid synthase	259	4.910331	1.01E−22	5.591387	4.95E−26
4	sp|Q15149|PLEC_HUMAN	PLEC	Plectin	220	1.397022	2.65E−22	1.404792	2.59E−18
5	sp|Q562R1|ACTBL_HUMAN	ACTBL2	Beta-actin-like protein 2	199	2.428919	0.272743016	2.635309	0.104194321
6	sp|Q7Z406|MYH14_HUMAN	MYH14	Myosin-14	176	5.295912	0.297084481	5.43472	0.193289608
7	tr|Q562M3|Q562M3_HUMAN	ACT	Actin-like protein (Fragment)	166	9.05212	0.372466028	9.267368	0.316949397
8	sp|Q13813|SPTN1_HUMAN	SPTAN1	Spectrin alpha chain, non-erythrocytic 1	156	1.911921	1.78E−30	2.049046	1.18E−29
9	sp|Q01082|SPTB2_HUMAN	SPTBN1	Spectrin beta chain, non-erythrocytic 1	129	1.72504	7.48E−17	1.836707	2.02E−19
10	sp|O43707|ACTN4_HUMAN	ACTN4	Alpha-actinin-4	127	2.037938	4.27E−11	2.040798	1.32E−08
11	tr|A0A024QZ63|A0A024QZ63_HUMAN	hCG_27198	HCG27198, isoform CRA_c	96	2.793359	1.11E−17	2.821251	1.73E−17
12	tr|E9PDF6|E9PDF6_HUMAN	MYO1B	Unconventional myosin-Ib	93	3.796812	3.36E−16	3.796442	2.41E−14
13	sp|P12814|ACTN1_HUMAN	ACTN1	Alpha-actinin-1	89	1.795801	1.21E−07	1.827206	4.69E−10
14	tr|E9PMS6|E9PMS6_HUMAN	LMO7	LIM domain only protein 7	80	5.813494	0.00171887	5.941699	0.000842874
15	sp|O00159|MYO1C_HUMAN	MYO1C	Unconventional myosin-Ic	79	3.791151	4.93E−15	4.197793	2.33E−16
16	sp|P05187|PPB1_HUMAN	ALPP	Alkaline phosphatase, placental type	77	3.799343	2.03E−07	3.740495	4.97E−06
17	tr|H6VRG3|H6VRG3_HUMAN	KRT1	Keratin 1	74	1.389211	4.01E−05	1.443922	9.06E−05
18	tr|Q6IBG1|Q6IBG1_HUMAN	MYL9	MYL9 protein	72	5.451808	0.100002006	5.182482	0.143713728
19	tr|E7ERU0|E7ERU0_HUMAN	DST	Dystonin	65	1.370127	5.16E−06	1.3949	0.000138176
20	tr|A0A024RAC0|A0A024RAC0_HUMAN	LUZP1	Leucine zipper protein 1, isoform CRA_a	55	2.472267	2.43E−09	2.691021	1.41E−10
21	sp|Q6WCQ1|MPRIP_HUMAN	MPRIP	Myosin phosphatase Rho-interacting protein	55	3.734554	2.18E−06	4.085938	2.84E−05
22	sp|P07951|TPM2_HUMAN	TPM2	Tropomyosin beta chain	53	2.661941	0.144164726	2.68936	0.13973473
23	tr|B2RMV2|B2RMV2_HUMAN	CYTSA	CYTSA protein	49	2.508032	3.31E−07	2.754671	4.77E−08
24	sp|Q14126|DSG2_HUMAN	DSG2	Desmoglein-2	48	1.883534	1.69E−06	2.019478	1.55E−07
25	sp|P13645|K1C10_HUMAN	KRT10	Keratin, type I cytoskeletal 10	48	1.602823	2.68E−05	1.690192	0.000321689
26	sp|Q13045|FLII_HUMAN	FLII	Protein flightless-1 homolog	47	2.873587	2.94E−05	2.817423	0.000103526
27	sp|P52907|CAZA1_HUMAN	CAPZA1	F-actin-capping protein subunit alpha-1	46	2.671351	7.88E−05	2.75277	4.64E−05
28	sp|Q16658|FSCN1_HUMAN	FSCN1	Fascin	43	1.463882	6.24E−06	1.533627	8.91E−05
29	sp|Q9NYL9|TMOD3_HUMAN	TMOD3	Tropomodulin-3	43	3.386163	1.76E−05	3.824799	4.70E−06
30	tr|A0A024R1X8|A0A024R1X8_HUMAN	JUP	Junction plakoglobin, isoform CRA_a	42	1.554556	3.78E−06	1.593763	2.35E−06
31	sp|O95425|SVIL_HUMAN	SVIL	Supervillin	41	2.602663	0.001037095	2.961881	0.000195296
32	tr|H0YNH8|H0YNH8_HUMAN	UACA	Uveal autoantigen with coiled-coil domains and ankyrin repeats	39	2.042608	3.15E−09	2.11368	1.46E−08
33	sp|P06753|TPM3_HUMAN	TPM3	Tropomyosin alpha-3 chain	39	4.380041		4.116189	
34	sp|P09493|TPM1_HUMAN	TPM1	Tropomyosin alpha-1 chain	38	1.762385	0.077036962	1.635907	0.105389036
35	sp|Q16643|DREB_HUMAN	DBN1	Drebrin	30	2.311505	9.75E−06	2.453286	4.25E−05
36	sp|Q9P0K7|RAI14_HUMAN	RAI14	Ankycorbin	28	2.148152	6.41E−05	2.3614	2.19E−06
37	sp|O15020|SPTN2_HUMAN	SPTBN2	Spectrin beta chain, non-erythrocytic 2	28	1.406392	0.009421338	1.503684	0.000358563
38	sp|Q9Y608|LRRF2_HUMAN	LRRFIP2	Leucine-rich repeat flightless-interacting protein 2	28	2.300938	5.18E−05	2.352907	3.49E−05
39	sp|P35908|K22E_HUMAN	KRT2	Keratin, type II cytoskeletal 2 epidermal	28	1.646153	0.000577865	1.690331	1.07E−05
40	tr|A0A087X0K9|A0A087X0K9_HUMAN	TJP1	Tight junction protein ZO-1	27	1.514412	3.06E−05	1.427997	0.000557715
41	tr|Q6IB58|Q6IB58_HUMAN	FLOT1	FLOT1 protein	25	1.631902	9.55E−05	1.920704	7.39E−08
42	sp|Q13428|TCOF_HUMAN	TCOF1	Treacle protein	24	1.642456	0.000107874	1.874915	2.29E−05
43	sp|Q96N67|DOCK7_HUMAN	DOCK7	Dedicator of cytokinesis protein 7	22	1.327668	0.065086178	1.601374	0.008569049
44	sp|Q96FS4|SIPA1_HUMAN	SIPA1	Signal-induced proliferation-associated protein 1	21	2.159191	0.000983656	2.413208	0.000588341
45	sp|Q12792|TWF1_HUMAN	TWF1	Twinfilin-1	21	1.768837	0.001737681	1.843505	0.000554159
46	sp|P07948|LYN_HUMAN	LYN	Tyrosine-protein kinase Lyn	21	2.997176		2.528003	
47	sp|Q9NQX4|MYO5C_HUMAN	MYO5C	Unconventional myosin-Vc	20	1.705114	0.01927116	1.751134	0.003605252
48	sp|Q6NYC8|PPR18_HUMAN	PPP1R18	Phostensin	19	2.694653	5.02E−05	2.681495	5.17E−05
49	tr|A2NJV5|A2NJV5_HUMAN	IGKV A18	Kappa light chain variable region (Fragment)	19	2.28389	0.240758419	2.556679	0.229388431
50	sp|Q96C19|EFHD2_HUMAN	EFHD2	EF-hand domain-containing protein D2	18	2.413872	0.000136473	2.746724	4.88E−05
51	sp|Q8IVT2|MISP_HUMAN	MISP	Mitotic interactor and substrate of PLK1	17	2.113082	1.34E−06	2.19576	4.56E−07
52	sp|O60237|MYPT2_HUMAN	PPP1R12B	Protein phosphatase 1 regulatory subunit 12B	17	1.666243	0.01486919	1.897841	0.006569633
53	sp|Q9BY89|K1671_HUMAN	KIAA1671	Uncharacterized protein KIAA1671	16	1.389878	0.003938706	1.380789	0.009779588
54	sp|Q8N556|AFAP1_HUMAN	AFAP1	Actin filament-associated protein 1	15	1.739136	0.000295575	1.771639	0.001590264
55	sp|P04899|GNAI2_HUMAN	GNAI2	Guanine nucleotide-binding protein G(i) subunit alpha-2	15	1.390716	0.050071925	1.580288	0.008337548
56	sp|Q96IZ0|PAWR_HUMAN	PAWR	PRKC apoptosis WT1 regulator protein	15	2.101999	0.002897741	2.172414	0.00319098
57	sp|Q14651|PLSI_HUMAN	PLS1	Plastin-1	15	1.491069	0.366374075	1.739802	0.002665657
58	sp|P08754|GNAI3_HUMAN	GNAI3	Guanine nucleotide-binding protein G(k) subunit alpha	14	1.465639	0.173583567	1.466131	0.174515322
59	tr|Q5T6N4|Q5T6N4_HUMAN	ABLIM1	Actin-binding LIM protein 1	13	1.546123	0.410703063	1.857376	0.177637875
60	sp|Q96PY5|FMNL2_HUMAN	FMNL2	Formin-like protein 2	11	1.317802	0.352339953	1.351134	0.216772318
61	sp|Q6YHK3|CD109_HUMAN	CD109	CD109 antigen	11	1.334029	0.081696793	1.348319	0.149591982
62	sp|P62879|GBB2_HUMAN	GNB2	Guanine nucleotide-binding protein G(I)/G(S)/G(T) subunit beta-2	11	1.316142	0.118086331	1.369466	0.071457863
63	sp|P28289|TMOD1_HUMAN	TMOD1	Tropomodulin-1	10	1.720154	0.042252187	1.918302	0.008569272
64	sp|O15230|LAMA5_HUMAN	LAMA5	Laminin subunit alpha-5	10	1.339791	0.117773414	1.57948	0.062586941
65	tr|Q9NZ23|Q9NZ23_HUMAN	YA61	Drug-sensitive protein 1	9	1.441324	0.045518585	1.324137	0.034476336
66	tr|Q53Z63|Q53Z63_HUMAN	NT5E	5′-nucleotidase	8	1.411206	0.006373217	1.383428	0.003122543
67	sp|Q96II8|LRCH3_HUMAN	LRCH3	Leucine-rich repeat and calponin homology domain-containing protein 3	8	1.706335	0.049031802	1.635396	0.055755418
68	tr|Q8TE01|Q8TE01_HUMAN	derp12	DERP12 (Dermal papilla derived protein 12)	7	1.828045	0.010396365	1.854288	0.004546963
69	sp|Q96SB3|NEB2_HUMAN	PPP1R9B	Neurabin-2	6	1.395569	0.015349443	1.600011	0.003934822
70	tr|E9PR17|E9PR17_HUMAN	CD59	CD59 glycoprotein	6	1.85682	0.06617903	1.491637	0.179792181
71	sp|Q9BQI0|AIF1L_HUMAN	AIF1L	Allograft inflammatory factor 1-like	6	2.31311	0.025139008	2.56662	0.090089194
72	sp|P98179|RBM3_HUMAN	RBM3	Putative RNA-binding protein 3	6	2.875242	0.285602093	2.315469	0.405406147
73	sp|Q96MG8|PCMD1_HUMAN	PCMTD1	Protein-l-isoaspartate *O*-methyltransferase domain-containing protein 1	5	1.607658	0.017886819	1.554777	0.019220859
74	sp|P19474|RO52_HUMAN	TRIM21	E3 ubiquitin-protein ligase TRIM21	3	2.483759	0.021963865	2.311108	0.095597863
75	sp|O95466|FMNL_HUMAN	FMNL1	Formin-like protein 1	3	2.878891	0.403065771	1.972982	0.319309711
76	sp|Q4KMQ1|TPRN_HUMAN	TPRN	Taperin	3	1.340113	0.33699739	1.311182	0.583454609
77	sp|O95396|MOCS3_HUMAN	MOCS3	Adenylyltransferase and sulfurtransferase MOCS3	3	2.160296	0.529036522	2.914927	0.466293573
78	tr|K7ELP0|K7ELP0_HUMAN	TPM4	Tropomyosin alpha-4 chain (Fragment)	3	3.685683	0.056339081	4.30786	0.11501646
79	tr|A0A024R2J9|A0A024R2J9_HUMAN	TBC1D5	TBC1 domain family, member 5, isoform CRA_b	2	1.3353		1.753758	

### GO term enrichment analysis of FASN-interacting proteins

**Fig. 3:**
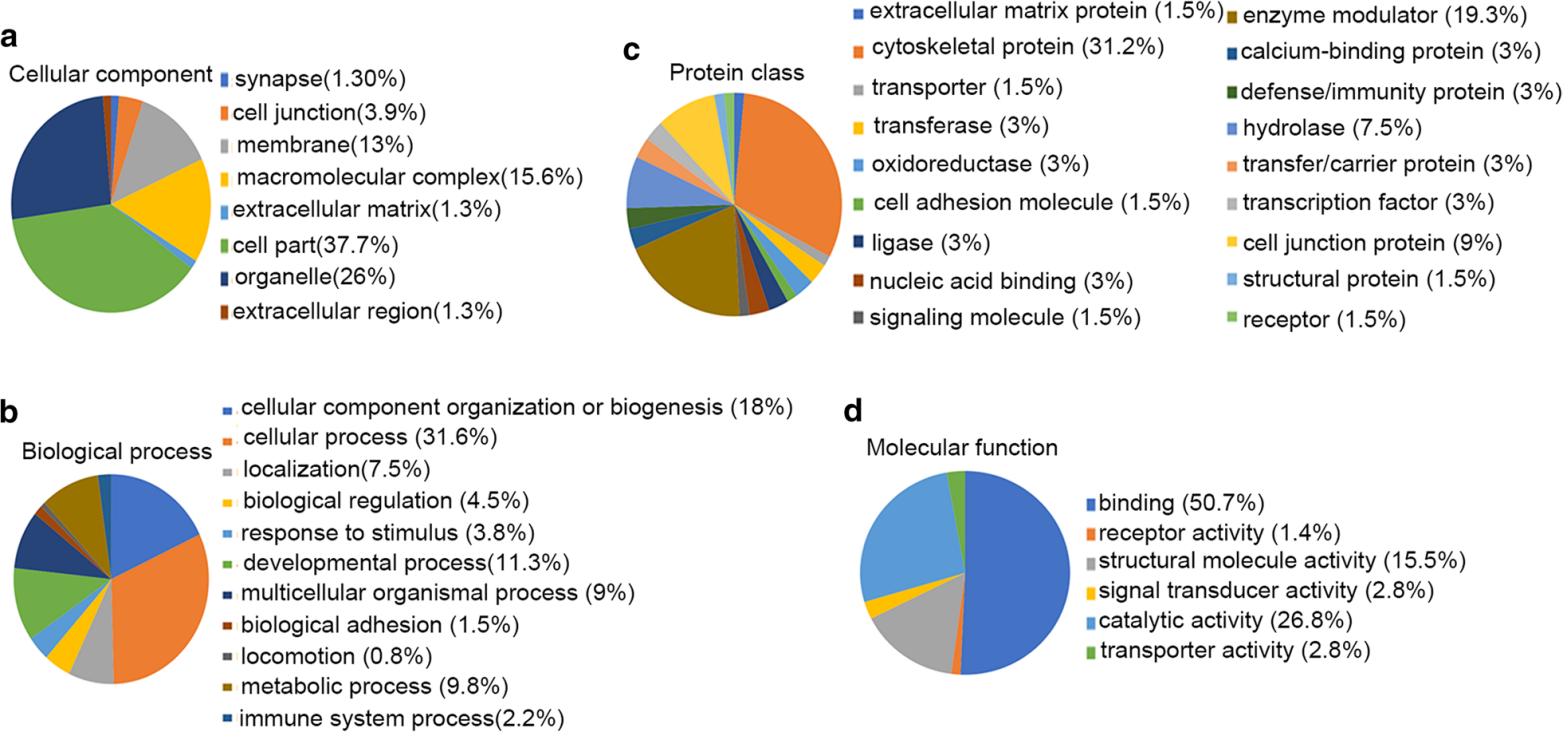
GO term enrichment analysis of proteins interacting with FASN performed using the PANTHER toolkit. The online software PANTHER was used to analyze **a** cellular components, **b** biological process, **c** protein class and **d** molecular function of FASN and proteins interacting with FASN. *GO* gene ontology, *FASN* fatty acid synthase

The online tools PANTHER was used to perform enrichment analysis of the 79 DEPs in order to identify the cellular components, biological processes, molecular functions and protein classes associated with FASN-interacting proteins. PANTHER analysis demonstrated that the enriched cellular components (Fig. 3a) mainly included ‘cell part’ and ‘macromolecular complex’. The enriched biological processes (Fig. 3b) mainly included ‘cellular process’ and ‘cellular component organization or biogenesis’. The enriched protein classes (Fig. 3c) mainly included ‘cytoskeletal protein’ and ‘cell junction protein’. The molecular functions analysis (Fig. 3d) revealed that the DEPs had ‘binding’ and ‘catalytic activity’. The online software WebGestalt revealed that the biological processes (Additional file 2: Fig. S2a) associated with the DEPs included ‘biological regulation’, ‘cellular component organization’, ‘metabolic process’, ‘localization’ and ‘cell proliferation’. The cellular components (Additional file 2: Fig. S2b) associated with the DEPs included ‘cytoskeleton’, ‘membrane’, ‘macromolecular complex’, ‘cytosol’, ‘nucleus’ and ‘cell projection’. In the analysis of DEP molecular functions (Additional file 2: Fig. S2c), ‘protein binding’, ‘ion binding’, ‘nucleotide binding’ and ‘structural molecule activity’ were the most common terms.

### Western blot analysis and immunofluorescence validation

**Fig. 4:**
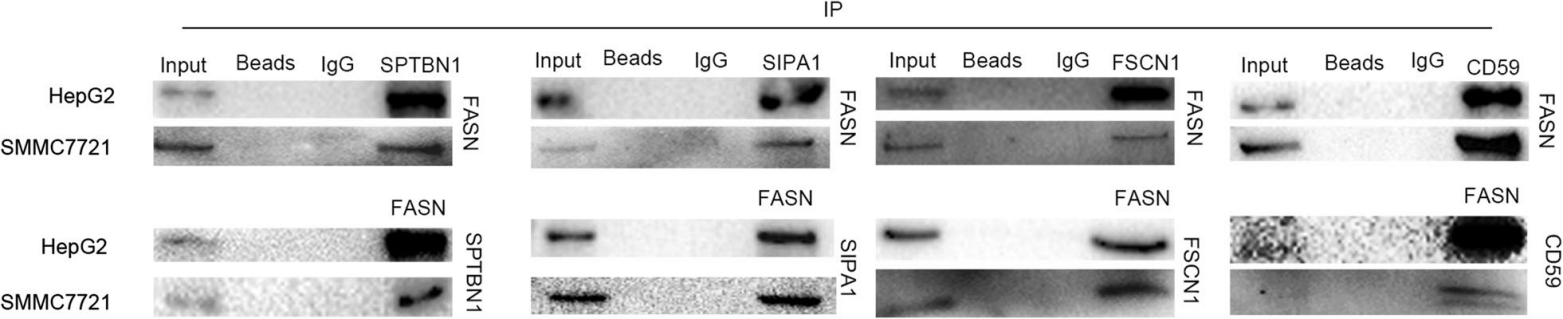
Co-IP was used to validate the iTRAQ results of proteins interacting with FASN. FASN-specific antibody was used to capture FASN-binding proteins. Normal rabbit IgG and agarose beads were used as a negative control in the hepatoma cell lines HepG2 and SMMC7721. All the co-IP and western blot analyses were performed at least three times. *Co-IP* co-immunoprecipitation, *iTRAQ* isobaric tags for relative and absolutely quantitation, *FASN* fatty acid synthase

**Fig. 5:**
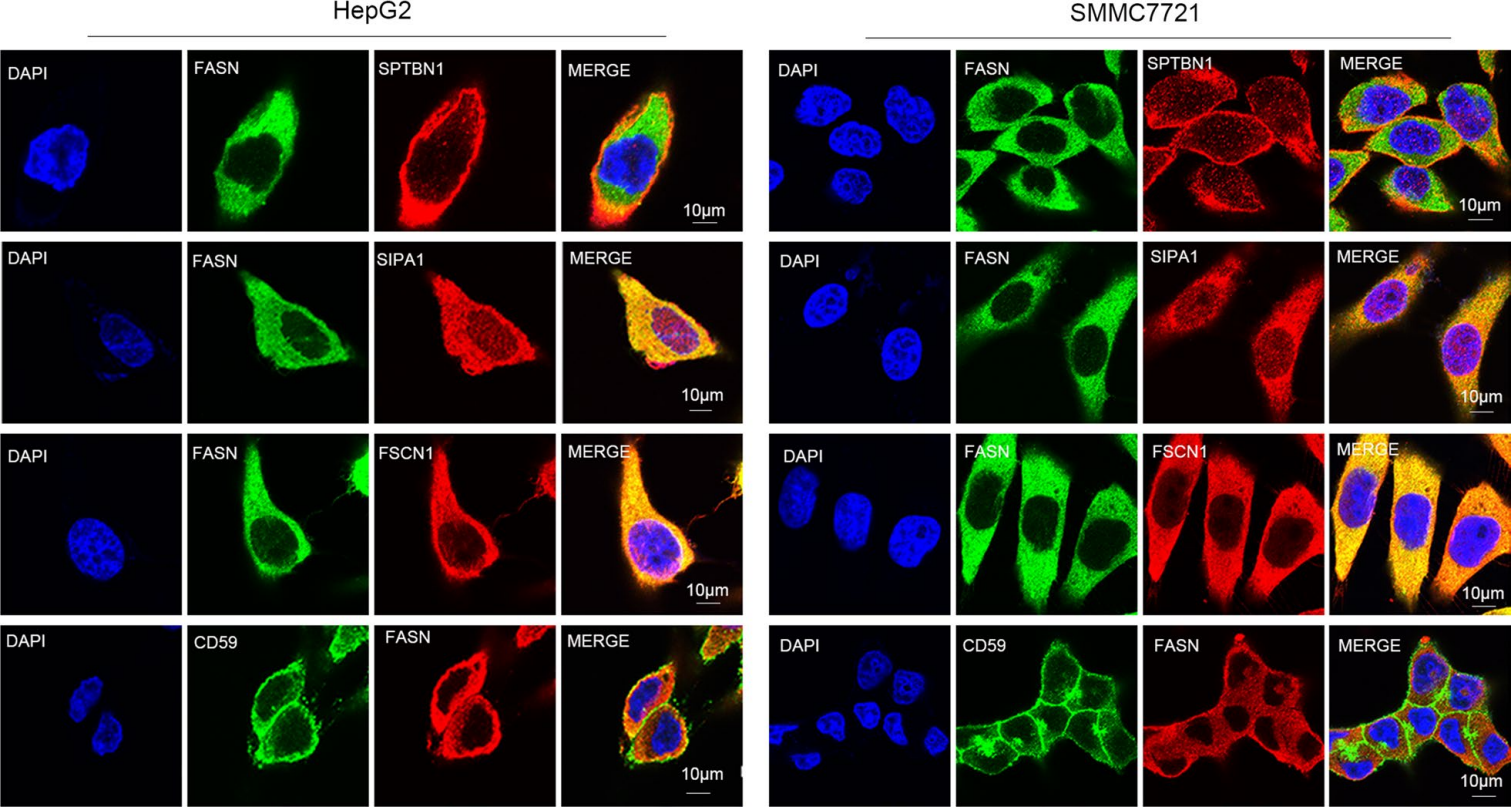
Co-localization of FASN and FASN-binding proteins. Hepatoma cells HepG2 and SMMC7721 were stained using antibodies against FASN, FSCN1, SIPA1, SPTBN1 and CD59, followed by incubation with FITC-conjugated donkey anti-rat or anti-mouse IgG. The cells were visualized using a confocal microscope. The yellow areas represent protein co-localization. Each experiment was performed in triplicate. *FASN* fatty acid synthase, *FSCN1* fascin actin-bundling protein 1, *SIPA1* signal-induced proliferation-associated 1, *SPTBN1* spectrin β, non-erythrocytic 1, *FITC* fluorescein isothiocyanate

The iTRAQ results demonstrated that there were 79 proteins FASN-interacting proteins and four of these proteins were selected for validation. Co-IP and western blot analyses were performed to verify the reliability of the iTRAQ results. FSCN1, signal-induced proliferation-associated 1 (SIPA1), SPTBN1 and CD59 were captured by co-IP with FASN used as the bait protein (Fig. 4). Confocal microscopy was used to observe the subcellular localization of FASN and its interacting proteins following immunostaining (Fig. 5).

### Effect of FASN knockdown on expression of FSCN1, SIPA1, SPTBN1 and CD59 in liver cancer cells

**Fig. 6:**
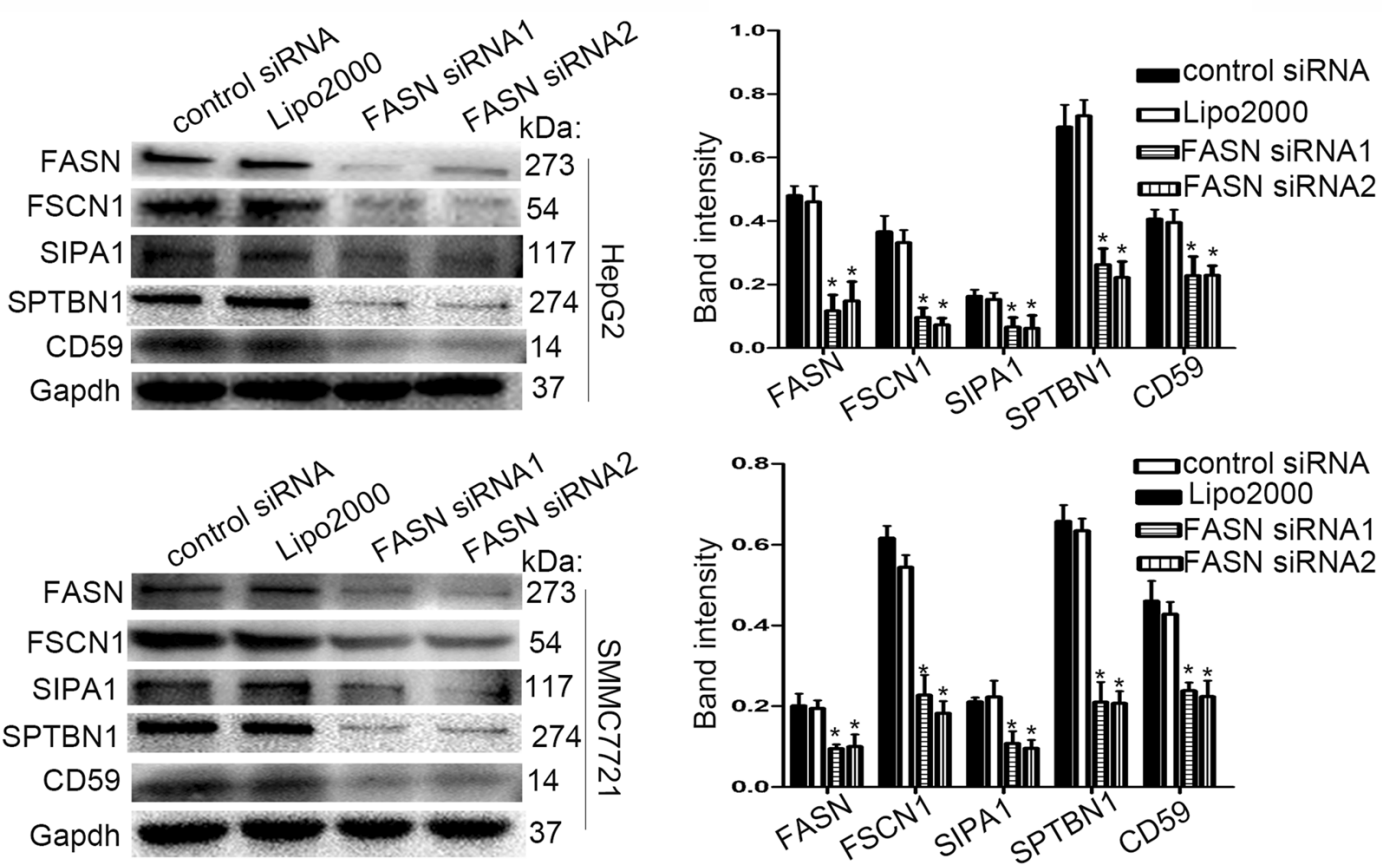
Effect of FASN knockdown on FSCN1, SIPA1, SPTBN1 and CD59 protein expression in liver cancer. The FASN siRNA significantly downregulated the expression of FASN in HepG2 and SMCC7721 cells and the expression levels of FSCN1, SIPA1, SPTBN1 and CD59 were significantly decreased in response to FASN silencing. The band intensity analysis of protein levels was performed using GAPDH as reference. All the western blot analyses were performed at least three times. *P < 0.05. Values are presented as the mean ± standard deviation. *FASN* fatty acid synthase, *FSCN1* fascin actin-bundling protein 1, *SIPA1* signal-induced proliferation-associated 1, *SPTBN1* spectrin β, non-erythrocytic 1, *siRNA* small interfering RNA

The results indicated that FASN was closely associated with liver cancer migration and invasion, and interacted with FSCN1, SIPA1, SPTBN1 and CD59. FASN siRNA was used to significantly downregulate the expression of FASN in HepG2 and SMCC7721 cells (Fig. 6), and the expression levels of FSCN1, SIPA1, SPTBN1 and CD59 were subsequently analyzed. As demonstrated in Fig. 6, the levels of these proteins were decreased by FASN knockdown, indicating that FASN may modulate the expression of these proteins to influence the progression of liver cancer.

### Effects of FASN, FSCN1 or SPTBN1 knockdown on MMPs in liver cancer

**Fig. 7:**
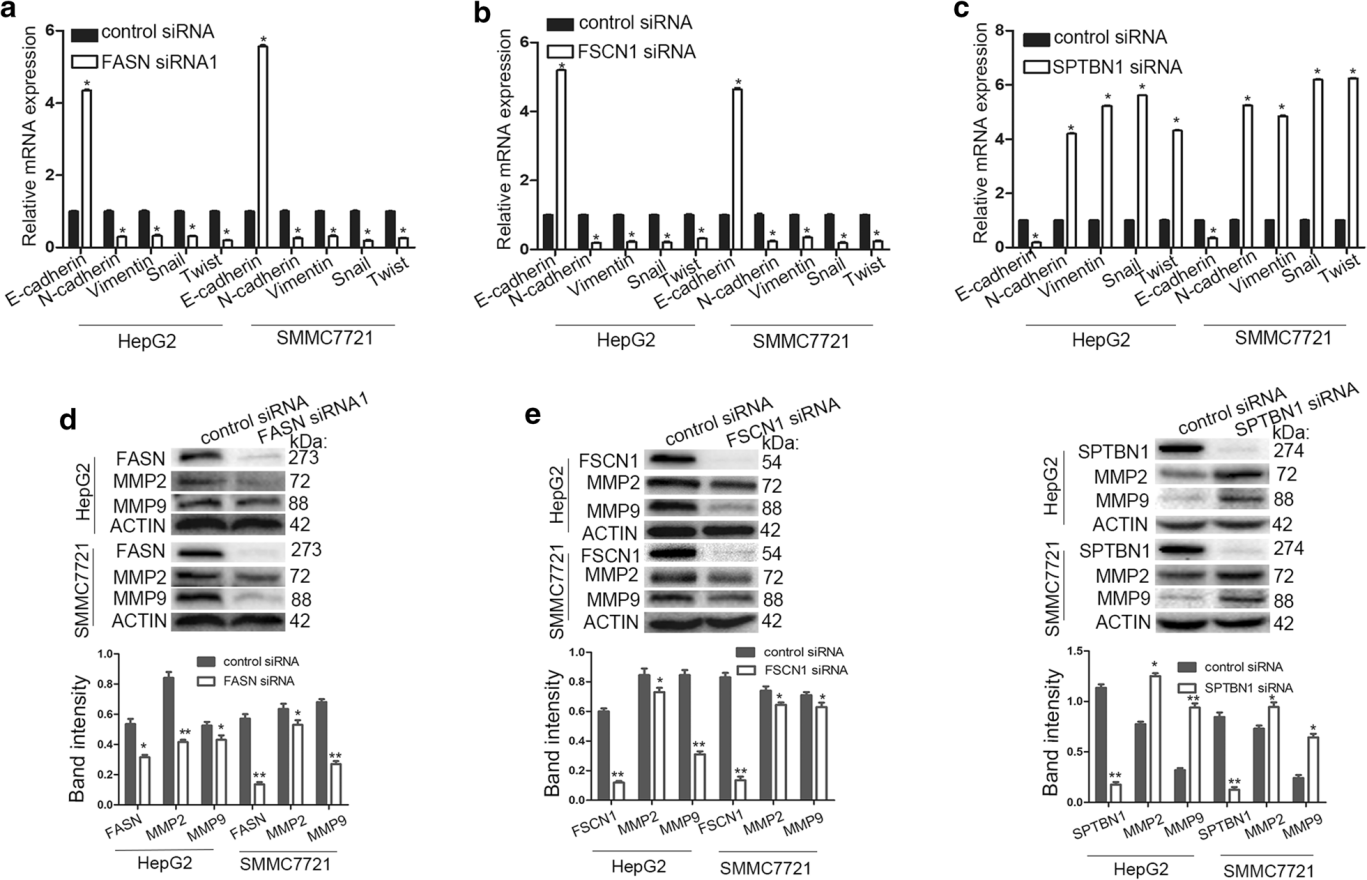
Effects of FASN, FSCN1 or SPTBN1 knockdown on EMT and MMPs in liver cancer. **a**–**c** mRNA expression of EMT-associated markers E-cadherin, N-cadherin, vimentin and transcription factors Snail and Twist were analyzed by reverse transcription-quantitative polymerase chain reaction. Knockdown of FASN or FSCN1 in HepG2 and SMCC7721 cells significantly decreased N-cadherin, vimentin, Snail and Twist, and increased E-cadherin expression, whereas knockdown of SPTBN1 produced the opposite results. **d**–**f** Western blot analyses were used to analyze the inhibition efficiency of FASN, FSCN1 or SPTBN1 siRNA. The protein expression of MMP-2 and MMP-9 in HepG2 and SMCC7721 cells were significantly reduced following silencing of FASN or FSCN1, whereas these proteins were increased following SPTBN1 knockdown. The band intensity analysis of protein levels was performed using actin as reference in western blot. Each experiment was performed in triplicate. *P < 0.05. Values are presented as the mean ± standard deviation. *FASN* fatty acid synthase, *FSCN1* fascin actin-bundling protein 1, *SPTBN1* spectrin β, non-erythrocytic 1, *EMT* epithelial–mesenchymal transition, *MMP* matrix metallopeptidase, *siRNA* small interfering RNA

As EMT and MMPs are closely associated with the increased migration and invasion capacity of tumor cells, EMT-associated markers, E-cadherin, N-cadherin, vimentin and transcription factors, Snail and Twist, were detected by RT-qPCR. MMP-2 and MMP-9 proteins were detected by western blot analysis. As demonstrated in Fig. 7a–c, knockdown of FASN or FSCN1 in HepG2 and SMCC7721 cells significantly decreased the mRNA expression of N-cadherin, vimentin, Snail and Twist, and increased E-cadherin; whereas, knockdown of SPTBN1 produced the opposite results. As demonstrated in Fig. 7d–f, MMP-2 and MMP-9 protein expression was significantly reduced in HepG2 and SMCC7721 cells following silencing of FASN or FSCN1; whereas, these proteins were increased following SPTBN1 knockdown.

## Discussion

Previous studies have demonstrated that FASN is associated with various human diseases, including obesity, inflammation, cardiovascular disease and cancer, in particular [25–28]. Overexpression of FASN is associated with disease progression and poor prognoses in a variety of malignant tumors, including prostate, breast and gastric cancer [12, 13]. The literature indicates that FASN promotes the proliferation, invasion and migration of tumor cells by interacting with various molecules, including nonstructural protein 5B and caveolin-1 [29, 30]. Therefore, FASN may be a promising therapeutic target used to reduce cancer progression and improve prognoses, and it is crucial to investigate the FASN protein interaction network in order to elucidate the molecular pathogenesis that drives cancer.

In our previous study, FASN was demonstrated to be upregulated in hepatocarcinoma and closely associated with the metastatic potential of liver cancer [18]. Additionally, wound healing and Transwell assay demonstrated that downregulation of FASN weakened liver cancer migration and invasion capacity. To clarify the molecular mechanism of FASN in the metastasis of liver cancer, FASN-interacting proteins were identified using targeted proteomics analyses (co-IP coupled with iTRAQ-based MS) of the hepatoma cell lines, HepG2 and SMMC7721. The biological functions of FASN-associated proteins were analyzed by bioinformatics methods, using the PANTHER and the WebGestalt toolkits. The analysis identified 79 FASN-interacting proteins using stringent filtering criteria. According to the bioinformatics analyses, FASN and the FASN-interacting proteins were associated with various biological process terms, including ‘cellular process’, ‘biological regulation’, ‘cellular component organization’, ‘multicellular organismal process’ and ‘metabolic process’ terms. These proteins were also associated with the protein classes, ‘cytoskeletal protein’ and ‘enzyme modulator’, and cellular components that the proteins were associated with included ‘membrane’, ‘macromolecular complex’ and ‘cell part’. With respect to the molecular functions, FASN and the FASN-interacting proteins were associated with ‘binding’, ‘catalytic activity’ and ‘structural molecule activity’. These results indicated that FASN and its interactome affect the development of liver cancer through various biological processes. To clarify the role of proteins interacting with FASN in liver cancer metastasis, four FASN-interacting proteins (FSCN1, SIPA1, SPTBN1 and CD59) were selected for validation.

FSCN1 is a 54-kDa, actin-binding protein required for the formation of cytoplasmic microfilament bundles and actin-based cell-surface protrusions [31]. FSCN1 is overexpressed in a variety of cancer types, including bladder and ovarian cancer [31, 32]. The overexpression of FSCN1 is commonly associated with distant metastasis, tumor progression, malignant infiltration and poor prognoses [32–34]. High expression levels of FSCN1 enhances cell kinetics and motility, increases the binding of β-catenin to cell boundaries, and regulates cell motility and adhesion [32, 35]. Additionally, FSCN1 may regulate nuclear factor-κB (NF-κB) activity, and the expression of MMP-2 and MMP-9, to promote tumor invasion and migration [35, 36]. SIPA1, a mitogen-inducible gene and a GTPase activating protein, is a negative regulator of Ras-related protein [37]. Overexpression of SIPA1 has been identified in several types of cancer, including colorectal and breast cancer [37, 38]. SIPA1 may have a key role in the invasion and metastasis of cancer via various signaling molecules and pathways. For instance, SIPA1 regulates the expression of MMP-7 and extracellular matrix-associated genes through interaction with bromodomain-containing protein 4 [38, 39]. SIPA1 can interact with the integrin β1 promoter and affect downstream focal adhesion kinase/PI3K/MMP-9 signaling [38]. In the current study, FSCN1 and SIPA1 were significantly downregulated following FASN knockdown in liver cancer cells. Furthermore, co-precipitation and co-localization of FASN with FSCN1 and SIPA1 in liver cancer indicated that FASN may mediate tumor metastasis via the PI3K/NF-κB/MMPs signaling pathway through interactions with FSCN1 or SIPA1.

SPTBN1 is an important TGF-β/mothers against decapentaplegic homolog (Smad) 3/4 adaptor protein and a transcriptional cofactor that regulates the TGF-β signaling pathway involved in many cellular processes, including cell proliferation, differentiation, apoptosis, migration and invasion [40, 41]. More recently, SPTBN1 has been reported to be abnormally expressed in several types of malignant tumor. Abnormal expression of FASN in the liver leads to cancer formation, and is associated with tumor progression and poor prognosis in liver cancer [42]. SPTBN1 may mediate liver cancer adhesive properties through an interaction with carcinoembryonic antigen related cell adhesion molecule 1**-**L and may have subsequent effects on the TGF-β-induced EMT signaling pathways [43]. SPTBN1 regulates molecular markers of EMT and the levels of the β-catenin target gene c-Myc via the Wnt signaling pathway, which mediates adhesion, migration and invasion of liver cancer [44]. CD59 is a widely distributed glycosylphosphatidylinositol-anchored protein that inhibits complement-mediated cell damage by preventing assembly of the membrane attack complex on host cells [45]. Recently, increasing research has demonstrated that CD59 is highly expressed in various forms of malignant tumor, including breast, prostate and gastrointestinal cancer, suggesting that CD59 is closely associated with tumor progression [46, 47]. CD59 has been reported to mediate proliferation, adhesion and migration of tumor cells through various signaling pathways. For instance, CD59 binding to Smad3 directly may promote invasion and migration in tumors via TGF-β-induced EMT [48]. The current study demonstrated that SPTBN1 and CD59 directly interact with FASN, and the protein expression levels of SPTBN1 and CD59 were decreased significantly by silencing of FASN. FASN may bind to SPTBN1 and CD59 to mediate invasion and migration in tumor cells, and regulate the activation of the TGF-β-induced EMT.

EMT is an important biological process induced by the c-met signaling pathway and is a crucial initiation step required for tumor migration and invasion [49]. MMPs, which are major proteolytic enzymes, have an important role in the degradation of the extracellular matrix, and thus, contribute to the regulation of tumor metastasis [49]. In the current study, silencing of FASN, FSCN1 or SPTBN1 expression in liver cancer cells led to changes in the mRNA expression of EMT-associated markers, E-cadherin, N-cadherin, vimentin and transcription factors Snail and Twist, and altered the protein expression of MMP-2 and MMP-9.

## Conclusion

In conclusion, iTRAQ-based proteomics analysis identified 79 proteins that interact with FASN. Four proteins (FSCN1, SIPA1, SPTBN1 or CD59) closely associated with tumor metastasis interacted with FASN and exhibited decreased expression in response to FASN silencing. Additionally, downregulation of FASN, FSCN1 or SPTBN1 resulted in altered expression of MMP-2, MMP-9 and EMT-associated proteins. Based on the functions of these proteins, it was concluded that FASN may bind these proteins to regulate invasion and metastasis in hepatocarcinoma, potentially by influencing EMT and MMPs; however, the specific mechanism remains unknown and requires further study.

## Supplementary information

**Additional file 1: Fig. S1.** Flow chart of the iTRAQ-based MS proteomics approach used in this study. iTRAQ, isobaric tags for relative and absolutely quantitation; MS, mass spectrometry.

**Additional file 2: Fig. S2.** GO term enrichment analysis of proteins interacting with FASN using the WebGestalt classification system. The online software WebGestalt was used to analyze (a) biological processes, (b) cellular components and (c) molecular functions of FASN, and proteins interacting with FASN. GO, gene ontology; FSCN1, fascin actin-bundling protein 1.

## Data Availability

The data supporting the conclusions of this paper are included within the manuscript.

## References

[1] Liu Z, Suo C, Mao X (2020). Global incidence trends in primary liver cancer by age at diagnosis, sex, region, and etiology, 1990–2017. Cancer.

[2] Ma X, Zhuang B, Li W (2017). MicroRNA-296-5p downregulated AKT2 to inhibit hepatocellular carcinoma cell proliferation, migration and invasion. Mol Med Rep.

[3] Torre LA, Bray F, Siegel RL (2015). Global cancer statistics, 2012. CA Cancer J Clin.

[4] Yang WS, Zeng XF, Liu ZN (2020). Diet and liver cancer risk: a narrative review of epidemiological evidence. Br J Nutr.

[5] Lv J, Zhang S, Wu H (2020). Deubiquitinase PSMD14 enhances hepatocellular carcinoma growth and metastasis by stabilizing GRB2. Cancer Lett.

[6] Omran NM, El-Sherbini SM, Hegazy O (2020). Crosstalk between miR‐215 and epithelial‐mesenchymal transition specific markers (E‐cadherin and N‐cadherin) in different stages of chronic HCV infection. J Med Virol.

[7] Guan Xiangming (2015). Cancer metastases: challenges and opportunities. Acta Pharm Sin B.

[8] Suhail Y, Cain MP, Vanaja K (2019). Systems biology of cancer metastasis. Cell Syst.

[9] Iadarola P (2019). Special issue: mass spectrometric proteomics. Molecules.

[10] O’Neill JR (2019). An overview of mass spectrometry-based methods for functional proteomics. Methods Mol Biol.

[11] Lupien LE, Dunkley EM, Maloy MJ (2019). An inhibitor of fatty acid synthase thioesterase domain with improved cytotoxicity against breast cancer cells and stability in plasma. J Pharmacol Exp Ther.

[12] Singh KB, Singh SV (2017). Fatty acid synthesis intermediates represent novel noninvasive biomarkers of prostate cancer chemoprevention by phenethyl isothiocyanate. Cancer Prev Res.

[13] Duan J, Sun L, Huang H (2016). Overexpression of fatty acid synthase predicts a poor prognosis for human gastric cancer. Mol Med Rep.

[14] Al-Bahlani S, Al-Lawati H, Al-Adawi M (2017). Fatty acid synthase regulates the chemosensitivity of breast cancer cells to cisplatin-induced apoptosis. Apoptosis.

[15] Liu ZL, Mao JH, Peng AF (2013). Inhibition of fatty acid synthase suppresses osteosarcoma cell invasion and migration via downregulation of the PI3K/Akt signaling pathway in vitro. Mol Med Rep.

[16] Jung MY, Kang JH, Hernandez DM (2018). Fatty acid synthase is required for profibrotic TGF-β signaling. FASEB J.

[17] Fiorentino M, Zadra G, Palescandolo E (2008). Overexpression of fatty acid synthase is associated with palmitoylation of Wnt1 and cytoplasmic stabilization of |[beta]|-catenin in prostate cancer. Lab Invest.

[18] Gong J, Shen S, Yang Y (2017). Inhibition of FASN suppresses migration, invasion and growth in hepatoma carcinoma cells by deregulating the HIF-1α/IGFBP1 pathway. Int J Oncol.

[19] Arkin M, Tang Y, Wells J (2014). Small-molecule inhibitors of protein–protein interactions: progressing toward the reality. Chem Biol.

[20] Ning Z, Hawley B, Chiang CK (2014). Detecting protein–protein interactions/complex components using mass spectrometry coupled techniques. Methods Mol Biol.

[21] Ghosh D, Li Z, Tan XF (2013). iTRAQ based quantitative proteomics approach validated the role of calcyclin binding protein (CacyBP) in promoting colorectal cancer metastasis. Mol Cell Proteom MCP.

[22] Liu T, Zhou J, Cui H (2018). iTRAQ-based quantitative proteomics reveals the neuroprotection of rhubarb in experimental intracerebral hemorrhage. J Ethnopharmacol.

[23] Zhang P, Zhu S, Li Y (2016). Quantitative proteomics analysis to identify diffuse axonal injury biomarkers in rats using iTRAQ coupled LC–MS/MS. J Proteom.

[24] Livak KJ, Schmittgen TD (2001). Analysis of relative gene expression data using real-time quantitative PCR and the 2(−Delta Delta C(T)) method. Methods.

[25] Wang D, DuBois RN (2012). Associations between obesity and cancer the role of fatty acid synthase.

[26] Angeles TS, Hudkins RL (2016). Recent advances in targeting the fatty acid biosynthetic pathway using fatty acid synthase inhibitors. Expert Opin Drug Discov.

[27] Bueno MJ, Quintela-Fandino M (2020). Emerging role of Fatty acid synthase in tumor initiation: implications for cancer prevention. Mol Cell Oncol.

[28] Buckley D, Duke G, Heuer TS (2017). Fatty acid synthase—modern tumor cell biology insights into a classical oncology target. Pharmacol Ther.

[29] Huang JT, Tseng CP, Liao MH (2013). Hepatitis C virus replication is modulated by the interaction of nonstructural protein NS5B and fatty acid synthase. J Virol.

[30] Karantanos T, Karanika S, Wang J (2016). Caveolin-1 regulates hormone resistance through lipid synthesis, creating novel therapeutic opportunities for castration-resistant prostate cancer. Oncotarget.

[31] Zhang N, Bi X, Zeng Y (2016). TGF-β1 promotes the migration and invasion of bladder carcinoma cells by increasing fascin1 expression. Oncol Rep.

[32] Park SH, Song JY, Kim YK (2014). Fascin1 expression in high-grade serous ovarian carcinoma is a prognostic marker and knockdown of fascin1 suppresses the proliferation of ovarian cancer cells. Int J Oncol.

[33] Guo L, Bai H, Zou D (2014). The role of microRNA-133b and its target gene FSCN1 in gastric cancer. J Exp Clin Cancer Res.

[34] Zhang M, Zhao Z, Duan X (2018). FSCN1 predicts survival and is regulated by a PI3K-dependent mechanism in renal cell carcinoma. J Cell Physiol.

[35] Liu C, Gao H, Cao L (2015). The role of FSCN1 in migration and invasion of pituitary adenomas. Mol Cell Endocrinol.

[36] Akanuma N, Hoshino I, Akutsu Y (2014). MicroRNA-133a regulates the mRNAs of two invadopodia-related proteins, FSCN1 and MMP14, in esophageal cancer. Br J Cancer.

[37] Ji K, Ye L, Toms AM (2012). Expression of signal-induced proliferation-associated gene 1 (SIPA1), a RapGTPase-activating protein, is increased in colorectal cancer and has diverse effects on functions of colorectal cancer cells. Cancer Genomics Proteomics.

[38] Zhang Y, Gong Y, Hu D (2015). Nuclear SIPA1 activates integrin β1 promoter and promotes invasion of breast cancer cells. Oncogene.

[39] Takahara T, Kasamatsu A, Yamatoji M (2017). SIPA1 promotes invasion and migration in human oral squamous cell carcinoma by ITGB1 and MMP7. Exp Cell Res.

[40] Horikoshi N, Pandita RK, Mujoo K (2016). β2-spectrin depletion impairs DNA damage repair. Oncotarget.

[41] Wang SY, Feng LY, Meng ZQ (2015). Bicluster and pathway enrichment analysis related to tumor progression of hepatocellular carcinoma. Eur Rev Med Pharmacol Sci.

[42] Baek HJ, Lee YM, Kim TH (2016). Caspase-3/7-mediated cleavage of β2-spectrin is required for acetaminophen-induced liver damage. Int J Biol Sci.

[43] Kiriyama S, Yokoyama S, Ueno M (2014). CEACAM1 long cytoplasmic domain isoform is associated with invasion and recurrence of hepatocellular carcinoma. Ann Surg Oncol.

[44] Zhi X, Lin L, Yang S (2015). βII-Spectrin (SPTBN1) suppresses progression of hepatocellular carcinoma and Wnt signaling by regulation of Wnt inhibitor kallistatin. Hepatology.

[45] Zhang R, Liu Q, Liao Q (2018). CD59: a promising target for tumor immunotherapy. Future Oncol.

[46] Ouyang Q, Zhang L, Jiang Y (2016). The membrane complement regulatory protein CD59 promotes tumor growth and predicts poor prognosis in breast cancer. Int J Oncol.

[47] Shang Y, Chai N, Gu Y (2014). Systematic immunohistochemical analysis of the expression of CD46, CD55, and CD59 in colon cancer. Arch Pathol Lab Med.

[48] Goswami MT, Kumar RA, Himabindu K (2016). Regulation of complement dependent cytotoxicity by TGF-β-induced epithelial–mesenchymal transition. Oncogene.

[49] Qi F, Wang J, Zhao L (2018). Cinobufacini inhibits epithelial–mesenchymal transition of human hepatocellular carcinoma cells through c-Met/ERK signaling pathway. Biosci Trends.

