# Supplementary figures and images for: Identification of the fatty acid synthase interaction network via iTRAQ-based proteomics indicates the potential molecular mechanisms of liver cancer metastasis
Source: Cancer Cell Int. 2020 Jul 21;20:332. doi: 10.1186/s12935-020-01409-2 (PMC7372886; doi:10.1186/s12935-020-01409-2)

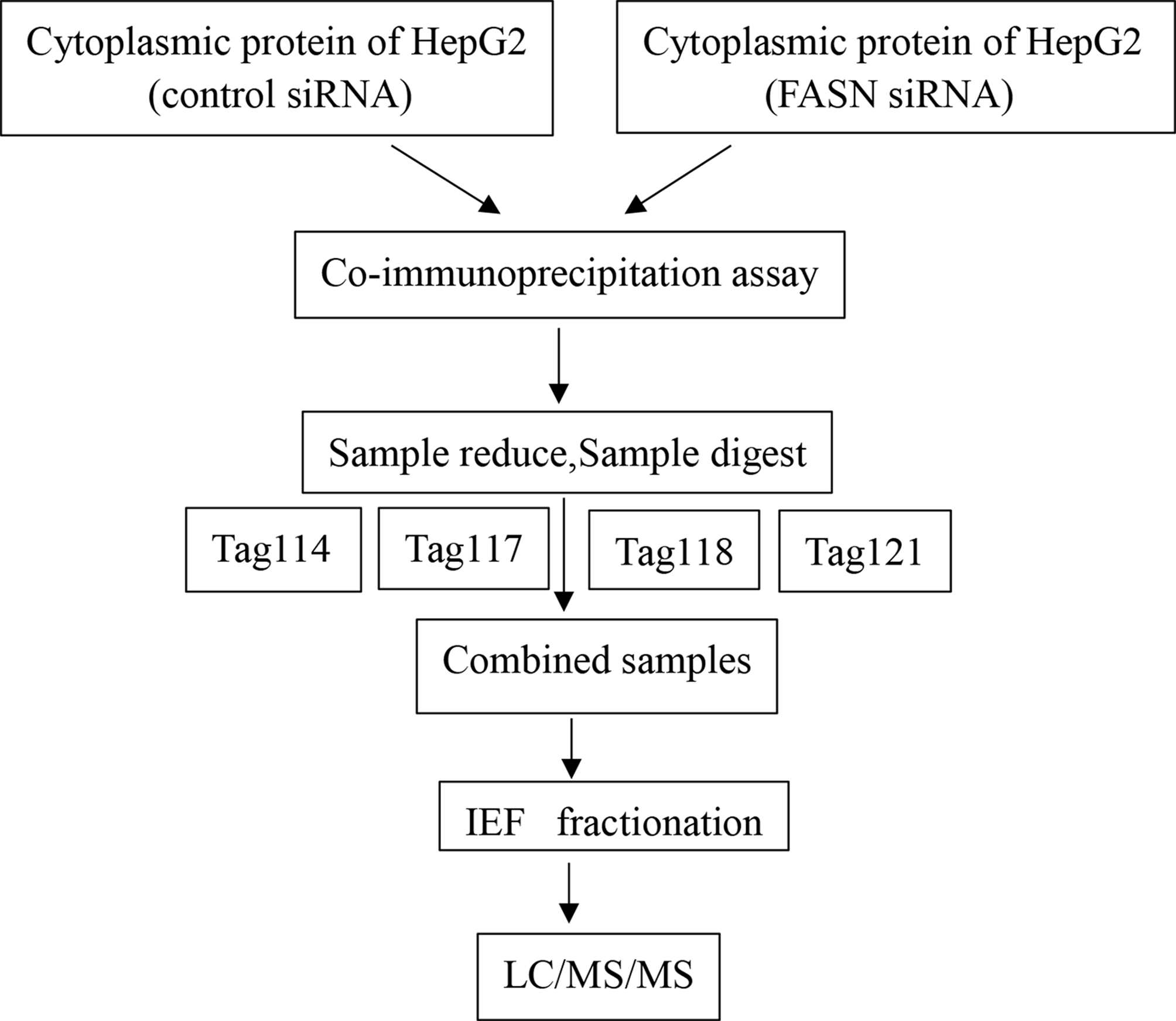

Supplement: Supplementary file 1 — Additional file 1: Fig. S1. Flow chart of the iTRAQ-based MS proteomics approach used in this study. iTRAQ, isobaric tags for relative and absolutely quantitation; MS, mass spectrometry. [file 12935_2020_1409_MOESM1_ESM.tif]

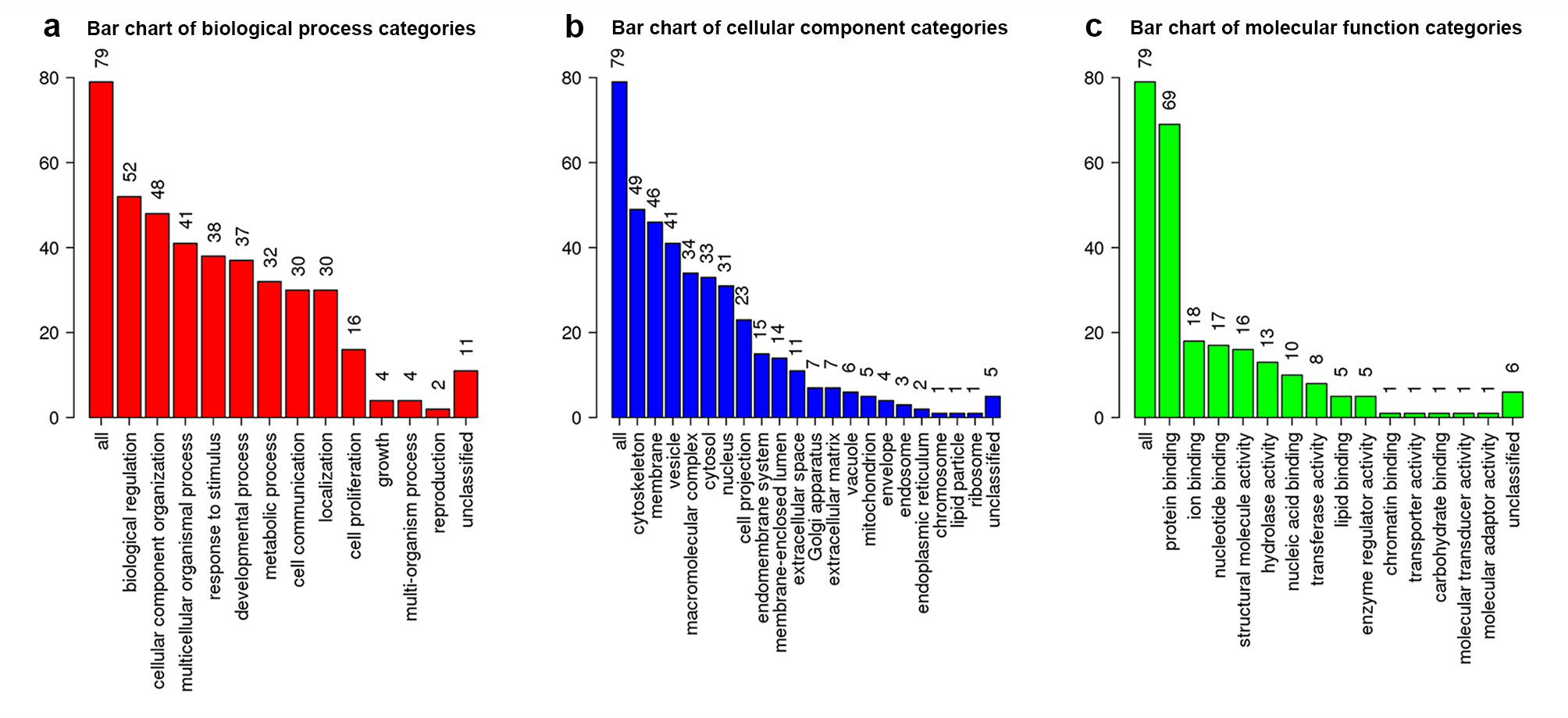

Supplement: Supplementary file 2 — Additional file 2: Fig. S2. GO term enrichment analysis of proteins interacting with FASN using the WebGestalt classification system. The online software WebGestalt was used to analyze (a) biological processes, (b) cellular components and (c) molecular functions of FASN, and proteins interacting with FASN. GO, gene ontology; FSCN1, fascin actin-bundling protein 1. [file 12935_2020_1409_MOESM2_ESM.tif]
